# Clean care for all—it’s in your hands: The May 5, 2019, World Health Organization’s “SAVE LIVES: Clean Your Hands” campaign

**DOI:** 10.1017/ice.2019.86

**Published:** 2019-06

**Authors:** Alexandra Peters, Tcheun Borzykowski, Ermira Tartari, Claire Kilpatrick, Safiah Hwai Chuen Mai, Benedetta Allegranzi, Didier Pittet

**Affiliations:** 1 Infection Control Program and WHO Collaborating Center on Patient Safety, University of Geneva Hospitals and Faculty of Medicine, Geneva, Switzerland; 2 Infection Prevention and Control Global Unit, Department of Service Delivery and Safety, World Health Organization, Geneva, Switzerland

Quality healthcare should be available to everyone. The World Health Organization’s (WHO’s) concept of universal health coverage (UHC) ^1^ embodies the urgent need for access to healthcare for all people around the world. In addition to access, the concept of UHC incorporates the critical element of the necessary quality of delivered healthcare services. Infection prevention and control (IPC), with hand hygiene as the most effective measure, is a practical and evidence-based approach with a demonstrable impact on quality of care and patient safety across all levels of the health system.

Each year, the WHO “SAVE LIVES: Clean Your Hands” campaign aims to bring people together in support of hand hygiene improvement globally on or around May 5^th^.^2^ This year’s theme for global annual hand hygiene day reflects a strong focus on providing clean care, equally protecting all patients and healthcare workers from infection and antimicrobial resistance transmission, across all countries, including in low-resource settings.

The WHO urges ministries of health, healthcare facility leaders, IPC leaders, healthcare workers, and patient advocacy groups to contribute to effective IPC action including hand hygiene as a cornerstone of quality in healthcare (Table 1). The WHO invites all healthcare facilities to join the 2019 WHO Global Survey on IPC and Hand Hygiene using 2 validated assessment tools: one for evaluating the core components of IPC program and the other for a deep dive into hand hygiene activities (https://www.who.int/infection-prevention/campaigns/ipc-global-survey-2019/en/).

**Table 1.** The May 5, 2019, World Health Organization “SAVE LIVES: Clean Your Hands” Campaign Calls to Action

Campaign Participants	Call to Action
Healthcare workers	“Champion clean care—It’s in your hands.”
IPC leaders	“Monitor infection prevention and control standards—Take action and improve practices.”
Healthcare facility leaders	“Is your facility up to WHO infection control and hand hygiene standards? Take part in the WHO survey 2019 and take action!”
Ministries of health	“Does your country meet infection prevention and control standards? Monitor and act to achieve quality universal health coverage.”
Patient advocacy groups	“Ask for clean care—It’s your right.”

Note. IPC, infection prevention and control; WHO, World Health Organization.

On a facility level, the use of these tools gives institutions a clear understanding of the strengths and weaknesses of their IPC and hand hygiene program and recommends concrete actions to address existing gaps. These tools allow institutions to improve their IPC practices and policies in concrete and measurable ways, at their own speed and in their own context. The surveys are anonymous, and global results will be made available using only aggregated data. Thus, facilities and ministries of health can commit fully to working on improving IPC and patient safety without fear of scrutiny or possible negative repercussions.

**Fig. 1.**
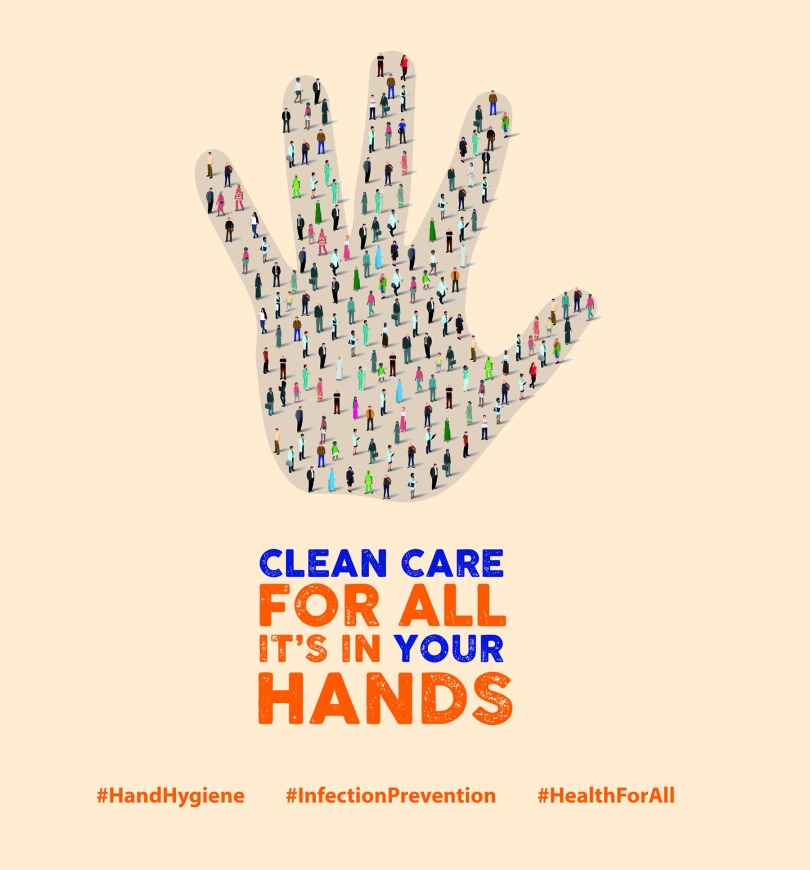
May 5, 2019: “Clean care for all—It’s in your hands!” The May 5, 2019, World Health Organization “SAVE LIVES: Clean Your Hands” campaign slogan and main promotional image (2019 hashtags: #HandHygiene #InfectionPrevention #HealthForAll). Campaign participants are invited to submit photos or selfies of them holding a board with the slogan and hashtags at www.CleanHandsSaveLives.org.

Globally, this survey will allow the WHO to provide a situational analysis of progress of current IPC and hand hygiene activities around the world and inform future efforts and resource use for IPC capacity building and improvement. Global surveys using the hand hygiene self-assessment framework were also conducted in 2011 and 2015,^3^ ^–^ ^5^ making this year’s survey even more crucial for tracking the implementation of hand hygiene and IPC on a global scale (Fig. 1).

Each improvement in IPC contributes toward quality UHC. “Clean care for all—it’s in your hands!”
